# *Campylobacter jejuni* Fatal Sepsis in a Patient with Non-Hodgkin’s Lymphoma: Case Report and Literature Review of a Difficult Diagnosis

**DOI:** 10.3390/ijms17040544

**Published:** 2016-04-12

**Authors:** Maria Teresa Gallo, Enea Gino Di Domenico, Luigi Toma, Francesco Marchesi, Lorella Pelagalli, Nicola Manghisi, Fiorentina Ascenzioni, Grazia Prignano, Andrea Mengarelli, Fabrizio Ensoli

**Affiliations:** 1 Department of Clinical Pathology and Microbiology, San Gallicano Institute, IRCCS, Rome 00144, Italy; gallo@ifo.it (M.T.G.); n.manghisi@gmail.com (N.M.); prignano@ifo.it (G.P.); ensoli@ifo.it (F.E.); 2 Department of Infectious Disease, San Gallicano Institute, IRCCS, Rome 00144, Italy; toma@ifo.it; 3 Department of Hematology, Regina Elena National Cancer Institute IRCCS, Rome 00144, Italy; marchesi.francesco@tiscali.it (F.M.); mengarelli@ifo.it (A.M.); 4 Intensive Care Medicine, Regina Elena National Cancer Institute IRCCS, Rome 00144, Italy; pelagalli@ifo.it; 5 Department of Biology and Biotechnology “Charles Darwin”, University of Rome Sapienza, Rome 00185, Italy; fiorentina.ascenzioni@uniroma1.it

**Keywords:** *Campylobacter jejuni*, non-Hodgkin’s lymphoma, chemotherapy, skin lesion

## Abstract

*Campylobacter jejuni* (*C. jejuni*) bacteremia is difficult to diagnose in individuals with hematological disorders undergoing chemotherapy. The cause can be attributed to the rarity of this infection, to the variable clinical presentation, and to the partial overlapping symptoms underlying the disease. Here, we report a case of a fatal sepsis caused by *C. jejuni* in a 76-year-old Caucasian man with non-Hodgkin’s lymphoma. After chemotherapeutic treatment, the patient experienced fever associated with severe neutropenia and thrombocytopenia without hemodynamic instability, abdominal pain, and diarrhea. The slow growth of *C. jejuni* in the blood culture systems and the difficulty in identifying it with conventional biochemical phenotyping methods contributed to the delay of administering a targeted antimicrobial treatment, leading to a fatal outcome. Early recognition and timely intervention are critical for the successful management of *C. jejuni* infection. Symptoms may be difficult to recognize in immunocompromised patients undergoing chemotherapy. Thus, it is important to increase physician awareness regarding the clinical manifestations of *C. jejuni* to improve therapeutic efficacy. Moreover, the use of more aggressive empirical antimicrobial treatments with aminoglycosides and/or carbapenems should be considered in immunosuppressed patients, in comparison to those currently indicated in the guidelines for cancer-related infections supporting the use of cephalosporins as monotherapy.

## 1. Introduction

*Campylobacter jejuni* represents one of the most common worldwide causes of bacterial gastroenteritis with over 190,000 cases occurring annually in the 27 member states of the European Union (www.efsa.europa.eu/efsajournal). Clinical manifestations include abdominal pain, fever, and diarrhea [1].

Unlike other enteric infections, *C. jejuni* is only rarely associated with extraintestinal localization and systemic invasive illness [1,2]. Bacteremia caused by *C. jejuni* has been detected in less than 1% of patients with gastroenteritis and it has been mainly reported in elderly and in immunocompromised patients [1,2].

In this study, we describe a case of *C. jejuni* sepsis in a patient with non-Hodgkin’s lymphoma that resulted in a fatal outcome. The low incidence of *C. jejuni* bacteremia and the paucity of associated symptoms make this infection difficult to detect in patients with hematological disorders where selecting the appropriate antibiotic treatment is crucial, and at present, early and distinctive clinical features have not yet been fully elucidated.

## 2. Case Presentation

A 76-year-old man was hospitalized in our Department of Hematology of the “Regina Elena” National Cancer Institute in Rome on 13 March, 2014. He suffered from a Diffuse Large B-Cell Lymphoma that had evolved from a previously diagnosed indolent non-Hodgkin Lymphoma (NHL) which was refractory to three chemo-immunotherapeutic lines of treatment and was characterized by cerebral and meningeal involvement at the time of last progression.

**Figure 1:**
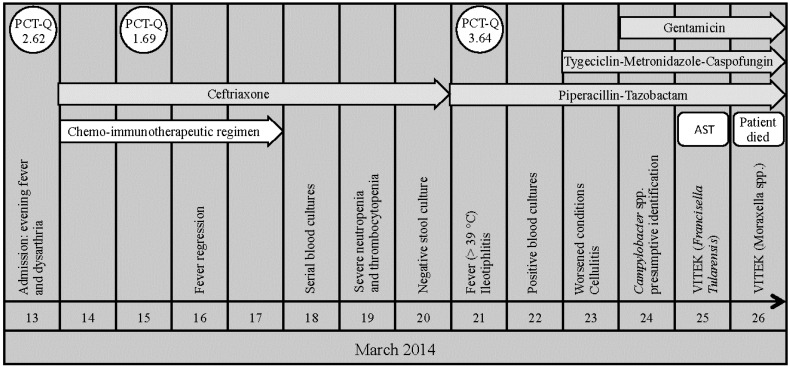
The patient’s clinical course. Procalcitonin (PCT-Q) levels were expressed as ng/mL. Antimicrobial susceptibility testing (AST) was performed by Etest^®^, according to the Clinical and Laboratory Standards Institute (CLSI) breakpoints for non-Enterobacteriaceae.

Upon admission, the patient had evening fever and severe dysarthria (Figure 1). On March 14, he received an urgent salvage treatment based on a chemo-immunotherapeutic regimen containing Rituximab 375 mg/m^2^ on day 1, Methotrexate 1 g/m^2^ on day 2, and Cytarabine 1 g total dose twice daily, for days 3 and 4. Given the presence of evening fevers and a moderate increase in procalcitonin levels (mini VIDAS system, bioMérieux, Florence, Italy) to 2.62 ng/mL (normal, <0.5 ng/mL), an empirical antibiotic therapy was administered including Ceftriaxone (2 g daily) at the beginning of the salvage chemo-immunotherapy, even in the absence of any microbiological evidence from the blood cultures and surveillance swabs. After 48 h, a complete regression of fever and a decrease in procalcitonin levels to 1.69 ng/mL were observed. Serial blood cultures, taken on March 18, were incubated in an automated, noninvasive culture system (BacT/ALERT, bioMérieux, Florence, Italy).

On March 19, the hemocytometric assessment showed severe neutropenia and thrombocytopenia (hemoglobin 75 g/liter, platelet count 6 × 10^9^/liter, white blood cell count 0.06 × 10^9^/liter). On March 20, the stool culture exam gave negative results. Nevertheless, on March 21, the patient had a relapse (fever > 39 °C) in the absence of symptoms indicating hemodynamic instability as well as abdominal pain or diarrhea. Based on the assumption that the patient was undergoing a sepsis, the patient was empirically treated with intravenous Piperacillin-Tazobactam (4.5 g three times a day), without clinical improvement. The abdominal echography revealed a severe circumferential thickening of the cecum wall with submucosal edema, whereas procalcitonin levels increased to 3.64 ng/mL. Meanwhile, on March 22, the blood cultures were positive revealing curved gram-negative rods at the microscopic analysis. The organism was subcultured onto chocolate agar (bioMérieux, Florence, Italy) and then incubated at 36 °C in a microaerophilic environment with 5% CO_2_. Thus, on March 23, based on the abdominal echography (suggestive for ileotiphlitis), and the patient’s general clinical conditions and increased procalcitonin levels, even in the absence of microbiological data (blood cultures were negative, so far), a different antibiotic therapeutic regimen was implemented. The patient was administered Tygeciclin 50 mg intravenously twice a day after a loading dose of 100 mg, Metronidazole 500 mg four times a day, and Caspofungin 50 mg daily after a loading dose of 70 mg. Despite implementing this type of antibiotic treatment, a rapid clinical deterioration in the patient was observed. Additionally, on March 23, cellulitis in the patient’s left leg was observed during a dermatological consultation. However, a skin biopsy was not advised due to the general health condition of the patient.

After 48 h of incubation, on March 24, irregular shaped grey and flat colonies appeared on the chocolate agar plates. The isolate was initially identified as *C. jejuni* by distinct colony morphology and by conventional biochemical tests resulting in oxidase- and hippurate-positive results.

Despite the microbiology laboratory promptly notifying the possible or likely infection of *C. jejuni* and the immediate implementation of empirical intravenous treatment with Gentamicin 6 mg/kg/Die, a further worsening of the patient’s clinical condition was observed on March 24. Surprisingly, microbiological testing by VITEK 2 system (bioMérieux, Florence, Italy) initially identified the microorganism as *Francisella Tularensis* (96% of identification confidence) whereas repeated testing yielded *Moraxella* spp. (95% of identification confidence), thereby creating uncertainty in the identification of the microorganism present. Thus, the poor health condition of the patient and severe cytopenia (hemoglobin 69 g/liter, platelet count 3 × 10^9^/liter, and white blood cell count 0.5 × 10^9^/liter) contributed to a rapid fatal outcome on March 26. Further identification of the microorganism was performed by sequence analysis (ABI PRISM 3130xl Genetic Analyzer) of the *16S rRNA* gene [3]. The sequence showed 99.9% similarity and 100% coverage for the strains of *C. jejuni* subsp. *jejuni* ATCC 700819. The sequences were deposited in the European Nucleotide Archive (ENA) with accession number LN864495.

Antimicrobial susceptibility testing (AST) was performed by Etest^®^, according to the Clinical and Laboratory Standards Institute (CLSI) breakpoints for non-Enterobacteriaceae as follows: ciprofloxacin, ≤1 μg/mL (Sensitive); doxycycline, ≤4 μg/mL (Sensitive); gentamicin, ≤4 μg/mL (Sensitive); meropenem, ≤4 μg/mL (Sensitive) (Table 1).

The Central Ethics Committee I.R.C.C.S. Lazio, section of the Istituti Fisioterapici Ospitalieri in Rome, in compliance with the Helsinki Declaration, approved this case report (Prot. CE/1016/15—4 December 2015).

Data and relevant scientific articles were identified via specific PubMed database searches from January 1980 and December 2015. The terms included in the search comprised: “Campylobacter jejuni” and “bacteremia” or “Campylobacter” and “bacteremia” or “non-Hodgkin’s lymphoma”. Research was restricted to English language articles.

## 3. Discussion

Infections caused by *C. jejuni* are only rarely complicated by extraintestinal localization or bacteremia [1]. In immunocompetent patients, *C. jejuni* bacteremia can be transient and resolved without antimicrobial therapy [1]. Conversely, individuals with immune deficiency or another serious underlying condition (cardiovascular disorders, hematological malignancies, liver disease, hypogammaglobulinemia, and human immunodeficiency virus infection) are exposed to an increased risk of bacteremia due to *C. jejuni* [2,4]. In these individuals, an effective antimicrobial treatment has been significantly associated with an improved outcome [2]. In a large number of cases, a timely identification of the pathogen and appropriate empirical antimicrobial therapy are hampered by the atypical presentation of the symptoms caused by *C. jejuni* [1,2]. The clinical signs of *Campylobacter* bacteraemia are generally accompanied by an acute-onset febrile illness of a transient nature with self-limiting enteritis. Nevertheless, in a large percentage of cases the clinical presentation of *Campylobacter* bacteraemia may show a febrile illness without gastrointestinal symptoms [4]. Other typical manifestations observed in severe sepsis caused by *Campylobacter* may include, skin lesions, cytopenia, and diarrhea, however, these symptoms also occur frequently in patients with aggressive lymphomas undergoing chemotherapy [2]. Moreover, the absence of consensus on the optimal antibiotic regimen and the lack of studies comparing different empirical treatments for *C. jejuni* bacteraemia make it difficult for the clinician to select an appropriate antimicrobial therapy. Different strategies were adopted, including fluoroquinolones (ciprofloxacin), macrolides (erythromycin), and aminoglycosides (gentamicin) [5].

Fluoroquinolones (e.g., ciprofloxacin) were largely used for the treatment of *Campylobacter* infection and, in general, are considered the drugs of choice for the empirical treatment of diarrheal illnesses [6,7,8]. *Campylobacter* and other organisms, such as *Salmonella* or *Shigella* species, were generally susceptible to fluoroquinolones, thus empirical treatment with these drugs is used without waiting for the stool culture results. However, since the early 1990s a growing number of fluoroquinolone-resistant *Campylobacter* strains have been registered in Asia as well as in several European countries. This increase of resistant strains is not only the result of the excessive use of these antimicrobials in clinical practice, but it is also the consequence of the use of fluoroquinolones in food producing animals and in veterinary species [9,10,11]. Thus, the possibility of fluoroquinolone-resistant strains must be considered in all cases of *Campylobacter* bacteraemia.

In the presence of confirmed *Campylobacter* infections, macrolides (erythromycin, or alternatively clarithromycin or azithromycin) represent the frontline agents [12], with tetracycline, doxycycline, and chloramphenicol considered alternative drugs [8]. However, recent evidence suggests that *Campylobacter* is also becoming increasingly resistant to macrolides, which represents a rising concern for public health [13]. The use of macrolides at subtherapeutic levels in chickens is considered a major factor influencing the emergence of resistant strains [13,14,15]. Thus, for serious systemic infections it has been demonstrated that aminoglycoside, gentamicin, or carbapenems are the most efficient antimicrobials [2,4,8,16,17].

In our case, the absence of clear clinical signs of a possible infection with enteric pathogens suggested that the patient be treated with ceftriaxone in accordance with the guidelines for cancer-related infections in immunosuppressed patients that support the use of cephalosporins in monotherapy [18]. Third-generation cephalosporins are largely used for the empirical treatment of community-acquired infectious diarrhea. However, these antimicrobial agents have not been proven effective for treating bacteremia due to *Campylobacter* species other than *Campylobacter fetus* [2,19]. Moreover, the use of third-generation cephalosporins and fluoroquinolones in the treatment of *Campylobacter* bacteraemia has shown poor prognosis and a high frequency of resistant strains has resulted in a general discouragement towards using this class of antibiotics [4,20], particularly in hospitals and communities with a high prevalence of extended-spectrum beta-lactamases (ESBLs)-producing bacteria.

After the first antimicrobial treatment, the patient presented neutropenia and fever, and therapy was then subsequently changed. In the absence of relevant microbiological data, the guidelines for the empirical therapy of febrile neutropenic cancer patients receiving chemotherapy recommend the use of pipercillin-tazobactam as first line monotherapy for the treatment of bloodstream infections [21]. However, *Campylobacter* isolates are not regularly susceptible to penicillins [22,23,24] and the b-lactamase enzyme found in *C. jejuni* is preferentially inhibited by clavulanic acid, but not by tazobactam or sulbactam [22,24]. In our case, only after having diagnosed sepsis caused by *C. jejuni*, the patient was empirically treated with gentamicin and the subsequent susceptibility drug profile indicated that this strain was in fact susceptible to this antimicrobial (Table 1). Nevertheless, the patient died because of complications due to a septic status and multiorgan failure. It is important to note that the treatment with gentamicin in this patient had started long after the appearance of initial enteric symptoms (diarrhea) and the first signs of sepsis. The delayed start of the targeted antimicrobial treatment was due to the difficulty in identifying *C. jejuni* bacteraemia, which, in turn, was the consequence of the very slow growth of this bacterium in standard automatic blood culture systems [25]. In fact, blood cultures are only rarely performed in patients presenting an apparently simple diarrhea symptom. This, as well as the slow growth of the characteristics of *C. jejuni* and the self-limited nature of this infection may represent a contributing cause to underestimating the real incidence of *C. jejuni* bacteraemia [26]. Additionally, the inability of the automated biochemical phenotyping system to promptly and correctly identify *C. jejuni* further deferred the recognition of the pathogen. The slow growth of *C. jejuni* in the BacT/ALERT and the repeated unsuccessful attempts in identifying the bacteria reported for the VITEK 2 system made the recognition of this pathogen particularly elusive. Indeed, previous studies have demonstrated that despite the Neisseria*-*Haemophilus (NH) identification card for VITEK 2 correctly identifying most *C. jejuni* ssp. *Jejuni*, misclassifications occur at a rate of more than 10% [27]. In this case, the diagnosis, and consequently the start of an appropriate therapy, was further delayed by the negative result of the stool cultures after the first episodes of diarrhea. Diarrheal illnesses in patients with neoplasia and immunosuppressive therapy are rarely perceived as a necessity to perform blood cultures, even when there is a fever present. On the other hand, it should be considered that blood stream infections caused by *C. jejuni* might occur without evidence of diarrhea, suggesting that this bacterium can access the intestinal mucosa without causing local inflammation [28].

A retrospective study suggested that a diagnostic clue for the presence of *C. jejuni* infection might be represented by leukopenia or thrombocytopenia, particularly when associated with an acute febrile diarrheal illness [29]. However, in neoplastic and immune suppressed patients, such as in our case, the marked cytopenia might be interpreted as a result of the immunosuppressed status of the patient who underwent a chemo-immunotherapeutic program.

In addition, three days before the fatal outcome, the patient also experienced the occurrence of cellulitis of the left leg. It has been reported that, although less recognized, skin lesions may represent a complication of *Campylobacter* bacteraemia that occurs particularly in patients with immune-related problems [30]. Again, the presence of NHL and chemotherapy made it difficult to recognize cellulitis as a sign of *C. jejuni* infection since lymphomas can be also characterized by an initial skin presentation [31].

## 4. Conclusions

In summary, although *C. jejuni* bacteraemia is uncommon, it may develop either primarily or secondarily from gastroenteritis, and thereby may represent a severe disease for immunocompromised individuals [30]. Occasionally, both NHLs and *C. jejuni* sepsis may intertwine; in cases such as this, it may create difficulties in being able to make a plain distinction between the root cause(s) of a patient’s symptoms. Many hematological disorders, especially lymphoid neoplasms, have a high risk for infection, thus when dealing with immunocompromised patients a septic disease should be suspected even in the presence of mild symptoms. In aggressive lymphoma, and in patients undergoing chemotherapy, fatigue, fever, diarrhea, as well as skin lesions and cytopenia may occur frequently, but these symptoms may occur also in severe sepsis caused by *C. jejuni*. Recognizing the early symptoms of a *C. jejuni* bacteraemia in hematological patients is key to initiate an effective antimicrobial therapy. From our experience, and from the data reported in the literature [2,4], blood cultures should always be performed in febrile patients with gastroenteritis. Therapy with appropriate antimicrobial agents is an important component in the management of immunocompromised patients with *C. jejuni* bacteraemia. Guidelines for cancer-related infections in immunosuppressed patients support the use of cephalosporins in monotherapy [18], whereas for the treatment of febrile neutropenic cancer patients receiving chemotherapy the use of pipercillin-tazobactam is recommended [21]. From our study, and from the data reported in the literature, it emerged that immunosuppressed patients with suspected *Campylobacter* sepsis should receive a more aggressive antimicrobial treatment—possibly combining aminoglycosides and/or carbapenems with cephalosporins in the first line antimicrobial empirical treatment. Nevertheless, the risk caused by the rise in antibiotic resistance among bacteria, particularly with *Campylobacter* spp. should also be considered where an increase in the administration of multiple antibiotics is likely to lead to colonization and infection with antibiotic-resistant organisms [32].

In this case, the unequivocal identification of *C. jejuni* was not obtained in time, and only by sequence analysis of the 16S rRNA gene. This further suggested that diagnostic systems, other than those based on the biochemical identification (*i.e.*, molecular techniques and Mass Spectrometry—MS) should be preferred for a prompt and unequivocal laboratory identification of *C. jejuni*. Combined molecular protocols (such as 16S rRNA PCR, DNA sequencing, and Multilocus Sequence Typing (MLST) analysis) revealed the successful identification of *C. jejuni* strains from stool and from blood cultures, even in patients where traditional culture protocols failed [33,34,35,36]. These results demonstrate the potential of molecular methods in improving the diagnosis of bacterial infections caused by *C. jejuni*. Numerous PCR-based techniques (real-time PCR and pyrosequencing) have also been developed for the rapid detection and identification of bacteria in clinical blood specimens [37]. Commercially available real-time PCR for the direct detection of bacteria in blood has been introduced [38], but the use of these tools has not become routine in clinical microbiology laboratories. Indeed, molecular techniques are rather costly, and require people with high levels of technical expertise, and therefore these techniques are consequently not suitable for routine identification, particularly in institutes with limited financial resources or in developing countries. Moreover, the high sensitivity of PCR-based methods and DNA sequencing that have the potential to detect all bacterial DNA present in a clinical sample may cause serious problems in clinical interpretation. Background levels of bacterial DNA might be detected in the blood of patients in the absence of any signs of bacteremia [39].

Matrix Assisted Laser Desorption Ionization Time-Of-Flight (MALDI-TOF) MS is a reliable tool for a rapid, precise, and cost-effective classification of a broad spectrum of bacteria and yeast [40]. MALDI-TOF MS analysis was in complete agreement with molecular tests identifying *C. jejuni* and *C. coli* [41]. Moreover, changes in protein biomarkers, such as those caused by an amino acid substitution, have been used to differentiate between *C. jejuni* ssp. *jejuni* and subsp. *doylei*, and to assess phylogenetic relationships in different isolates [42]. Several studies have evaluated the contribution of MALDI-TOF MS towards identifying microorganisms in positive blood culture [40,43]. Results showed that MALDI-TOF MS accurately identified blood-borne organisms in more than 80% of cases. Nevertheless, the ability of MALDI-TOF MS to correctly identify microorganisms in blood cultures clearly depends on the bacteria concentration [44,45]. Novel application of MALDI-TOF MS has increased its potential in the detection of blood-borne organisms and thus may allow faster bacterial identification than the conventional automated blood cultures systems in the near future [46]. However, the efficacy of MALDI-TOS MS technology in reducing the time for identifying positive blood cultures, particularly for slow growing bacteria such as *C. jejuni*, remains to be evaluated.

Since individuals with hematological disorders, especially lymphoid neoplasms, have a high risk for infection, the close cooperation between the hematologist, infectious disease specialist, and microbiologist can be of primary importance in providing a timely and effective intervention.

## Figures and Tables

**Table 1:** Antibiotic susceptibility testing of the isolated bacteria.

Antibiotic Tested	MIC	Test Result
Ciprofloxacin	≤1 μg/mL	Sensitive
Doxycycline	≤4 mcg/mL	Sensitive
Gentamicin	≤4 mcg/mL	Sensitive
Meropenem	≤4 mcg/mL	Sensitive

MIC: Minimal Inhibitory Concentration performed by Etest^®^ (bioMérieux, Florence, Italy), according to the Clinical and Laboratory Standards Institute (CLSI) breakpoints for non-Enterobacteriaceae.
